# Enhancement by thyroxine of gastric carcinogenesis induced by N-methyl-N'-nitro-N-nitrosoguanidine in Wistar rats.

**DOI:** 10.1038/bjc.1993.378

**Published:** 1993-09

**Authors:** H. Iishi, M. Tatsuta, M. Baba, R. Yamamoto, H. Taniguchi

**Affiliations:** Department of Gastrointestinal Oncology, Center for Adult Diseases, Osaka, Japan.

## Abstract

**Images:**


 © Macmillan Press Ltd., 1993

### Enhancement by thyroxine of gastric carcinogenesis induced by N-methyl-N′-nitro-N-nitrosoguanidine in Wistar rats

H. Iishi[1,3], M. Tatsuta[1], M. Baba[1], R. Yamamoto[1] & H. Taniguchi[2]

*Departments of [1]Gastrointestinal Oncology and [2]Pathology, The Center for Adult Diseases, Osaka, 3-3, Nakamichi 1-chome, Higashinari-ku, Osaka 537, Japan.*

**Summary** The affects of L-thyroxine ($T_4$) on the incidence and histology of gastric cancers induced by N-methyl-N′-nitro-N-nitrosoguanidine (MNNG), and on the labelling index of gastric mucosal epithelial cells were investigated in Wistar rats. After oral treatment with MNNG for 25 weeks, the rats received s.c. injections of $T_4$ (0.2 $\mu g\ kg^{-1}$) in depot form every other day until the end of the experiment in Week 52. This long-term treatment with $T_4$ significantly increased the incidence of gastric cancers in Week 52. However, it did not influence the histological type of the gastric cancers. It also caused significant increases in the labelling indices of the fundic and antral epithelial cells. These findings indicate that $T_4$ enhances the development of gastric cancers, and that its effect may be related to its effect in increasing proliferation of gastric epithelial cells.

Thyroid hormones have important regulatory roles in the morphology and biochemistry of gastrointestinal mucosal cells (Hernandez *et al.*, 1988). Long-term $T_4$ administration increased the mitotic activity of the fundic 'stem' cells of the stomach and basal and secretagogue-stimulated acid secretion *via* its effect on parietal cell mass (Adeniyi & Olowookorun, 1989). We recently found that prolonged administration of $T_4$ significantly increased the incidence of rat colon tumours induced by azoxymethane (Iishi *et al.*, 1992). These findings suggest that $T_4$ might influence gastric carcinogenesis. Therefore, in the present work, we examined the effect of treatment of rats with $T_4$ on the development of gastric cancers in rats.

### Materials and methods

### *Animals*

Sixty 6-week-old male Wistar rats (SLC, Shizuoka, Japan) were used in this study. They were housed in stainless steel suspended wire mesh cages, under controlled environmental conditions of 12 h light and 12 h darkness, 30–50% humidity, and 21–22°C. Each rat was given standard laboratory chow (Oriental Yeast, Tokyo, Japan) at 60 kcal per day.

### *Experimental design*

The animals were given drinking water containing MNNG (25 $\mu g\ ml^{-1}$; Aldrich, Milwaukee, WI) for 25 weeks. The stock solution of MNNG was prepared at 1 $mg\ ml^{-1}$ in deionised water and kept in a cool, dark place and renewed every week. Just before administration to rats it was diluted to 25 $\mu g\ ml^{-1}$ with tap water. Rats were given 20 ml per day of MNNG solution each, supplied from bottles covered with aluminium foil to prevent photolysis of MNNG, and the solution was renewed every other day. Safety precautions were taken in use of MNNG. From Week 26, the rats were given normal tap water *ad libitum* and were randomly divided into two groups of 30 rats each. Group 1 was given s.c. injections of the vehicle, plain olive oil only, while Group 2 was given s.c. injections of $T_4$ in depot form (Sigma, St. Louis, MO, USA; 0.2 $\mu g\ kg^{-1}$ body weight) in olive oil until Week 52. Injections were given at various sites every other day in a volume of 1 $ml\ kg^{-1}$ body weight between 2 and 3 p.m.

Correspondence: H. Iishi.
Received 29 September 1992; and in revised form 20 April 1993.

### *Tissue sampling*

Animals that survived for more than 49 weeks were included in effective numbers, because the first tumour of the glandular stomach was found in a rat in Group 2 that died in Week 49. All surviving animals were killed at the end of the experiment in Week 52. All rats were autopsied, and the stomach and other organs were carefully examined. The stomach was opened along the greater curvature, pinned flat on a cork mat, and fixed in Zamboni's solution (Stefanini *et al.*, 1967) for histological examination. The fixed stomach was cut into longitudinal, 3 mm wide strips. The specimens were embedded in paraffin, and 5 $\mu m$ thick serial sections were stained with hematoxylin and eosin. Sections were examined without knowledge of which group they were from.

### *Classification of gastric cancers*

Histologically, adenocarcinomas were defined as lesions in which neoplastic cells had penetrated the muscularis mucosae to the submucosa or deeper layers. Adenocarcinomas were classified as very well-differentiated, well-differentiated, and poorly differentiated, as reported previously (Tatsuta *et al.*, 1988*b*). Very well-differentiated adenocarcinoma: cancers showing atypical glandular structure with a tubular or papillary pattern and an arrangement of cells comparable to that enclosing normal gastrointestinal crypts (Figure 1a). Well-differentiated adenocarcinoma: common type, less differentiated glands consisting of disorderly masses of atypical cells containing a small amount of intracellular mucin (Figure 1b); mucinous carcinoma, active mucin secretion, often resulting in mucinous nodules with a large amount of extracellular mucin, with only a few isolated groups of tumour cells (Figure 1c). Poorly differentiated adenocarcinoma: anaplastic carcinoma, highly anaplastic cells scattered individually with no typical glandular or tubular differentiation; signet-ring cell carcinoma, tumour cells with a large amount of intracellular mucin, giving the cells a signet-ring appearance (Figure 1d).

### *Measurement of labelling index of gastric mucosa*

Five rats in each group were killed in experimental Weeks 30 and 52 to determine the labelling index of the gastric mucosa with an immunohistochemical analysis kit (Becton-Dickinson, Mountain View, CA) for assay of bromodeoxyuridine (BrdU) incorporation (Gratzner, 1982; Morstyn *et al.*, 1983). For this purpose, the rats were starved for 12 h and then received s.c. injection of either 1 $ml\ kg^{-1}$ of olive oil (Group 1) or 0.2 $\mu g\ kg^{-1}$ of $T_4$ (Group 2). One hour later, the animals received an i.p. injection of BrdU (20 mg $kg^{-1}$), and after another hour were killed with ether. The stomach was removed and fixed in 70% ethanol for 4 h. Sections of 3 $\mu$m thickness were immersed in 2 N HCl solution for 30 min at room temperature, and then in 0.1 M $Na_2B_4O_7$ to neutralise the acid. The sections were then stained with anti-BrdU monoclonal antibody (diluted 1:100) for 2 h at room temperature, washed, treated with biotin-conjugated horse anti-mouse antibody (at 1:200 dilution) for 30 min, and stained with avidin-biotin-peroxidase complex for 30 min. The reaction product was localised with 3,3′-diaminobenzidine tetrahydrochloride. Cells containing BrdU were identified by the presence of dark pigment over their nucleus.

The labelling index of the gastric mucosa were determined by counting BrdU-labelled and unlabelled cells in the zone of proliferating cells (Eastwood & Quimby, 1983) without knowledge of which group the sample was from. The zone of proliferating cells in the fundic mucosa was defined as a 250-$\mu$m rectangular area between the highest and lowest labelled cells in well-oriented sections. Ten such rectangular areas in each rat were examined. In the antral mucosa, all cells below the highest labelled cells in each pit-gland column were regarded as being within the zone of proliferating cells. In each rat, 100 well-oriented columns of pits and glands were examined, and the labelling index was calculated as the number of BrdU-labelled cells/total number of cells within the zone of proliferating cells.

### *Measurements of serum $T_4$ and $T_3$*

Serum $T_4$ and triiodothyronine ($T_3$) were measured in Weeks 30 and 52. For this purpose, five rats in each group were kept for 12 h without food, and then received an s.c. injection of either 1 ml $kg^{-1}$ of olive oil (Group 1) or 0.2 $\mu g\ kg^{-1}$ of $T_4$ (Group 2). Two hours later, they were anesthetised with ether and blood was obtained by cardiac puncture. The serum was separated and stored at − 20°C. Within 1 week, the serum $T_4$ and $T_3$ were assayed with commercial radioimmunoassay kits (Gamma Coat $T_4$ RIA kit and Gamma Coat $T_3$ RIA kit).

### *Statistical analysis*

Incidence and distribution of the different histological types of gastric cancers were analysed by the chi-square test or Fisher's exact probability test (Siegel, 1956). Other results were all analysed by one-way analysis of variance with Dunn's multiple comparison (Snedecor & Cochran, 1967; Miller, 1966). Data are shown as mean ± s.e.m. 'Significant' indicates a calculated $P$ value of less than 0.05.

### Results

### *Incidence and histological type of gastric cancer*

The body weight, food consumption and incidence, numbers and histological types of gastric cancers in each group are summarised in Table I. In Week 52, animals that received $T_4$ (Group 2) had significantly lower body weights than untreated rats. Table I also shows that there were no significant differences between the food consumption in the two groups in Weeks 30 and 45. In Group 1 (olive oil), four gastric cancers were found in four (20%) of 20 rats examined. In Group 2 ($T_4$), 12 gastric cancers were found in 11 (55%) of 20 rats examined. The incidence of gastric cancers in Group 2 was significantly greater than that in Group 1. All the tumours induced in the glandular stomach were identified histologically as adenocarcinomas. Almost all were very well-differentiated, and neither mucinous carcinomas or poorly differentiated cancers were found in this series. There was no significant difference in the histological types of adenocarcinomas in the two groups: all cancers in Group 1 were very well-differentiated, while in Group 2 very well-differentiated adenocarcinomas were found in 11 (92%) of 12 tumours and the other was well-differentiated. All cancers were found in the antral mucosa, and no metastases were detected at the macroscopic and/or microscopic level.

### *Labelling index of gastric mucosa and serum $T_4$ and $T_3$ levels*

Table II summarises data on the labelling indices of gastric mucosa in Weeks 30 and 52. At both times, Group 2 ($T_4$) showed significantly higher labelling indices and increased number of cells in the zone of proliferating cells in both the fundic and antral mucosa than Group 1 (olive oil). Table III shows that at both times, administration of $T_4$ significantly increased the serum levels of $T_4$ and $T_3$.

### Discussion

In the present work, we found that $T_4$ enhanced gastric carcinogenesis induced by MNNG in Wistar rats. Long-term s.c. administration of $T_4$ in depot form significantly increased the incidence of gastric cancer, but had no influence on their histological type at autopsy in Week 52.

**Table I** Incidences and numbers of gastric cancers in MNNG-treated rats

| *Group no.* | *Treatment*[a] | *Body weight (g) 26W* | *52W* | *Food intake (g/day) 30W* | *45W* | *Effective no. of rats* | *No. of rats with gastric cancer (%)* | *No. of gastric cancers* |
|---|---|---|---|---|---|---|---|---|
| 1 | Olive oil | 319 ± 4 | 379 ± 6 | 21 ± 1 | 21 ± 1 | 20 | 4 (20) | 4 |
| 2 | $T_4$ | 320 ± 5 | 331 ± 5[c] | 19 ± 1 | 20 ± 1 | 20 | 11 (55)[b] | 12 |

[a]Treatment: Rats were given drinking water containing MNNG for 25 weeks, and then received s.c. injections of the vehicle, olive oil (Group 1) or 0.2 $\mu g\ kg^{-1}$ of thyroxine ($T_4$) in depot form (Group 2) every other day until the end of the experiment in week 52. [b,c]Significance of difference from the value in Group 1: [b]$P < 0.05$, [c]$P < 0.001$.

**Table II** Epithelial proliferation of gastric mucosa in MNNG-treated rats

| *Experimental week* | *Group no.* | *Treatment*[a] | *Fundic mucosa No. of labelled cells* | *No. of cells in proliferating zone* | *Labelling index (%)* | *Antral mucosa No. of labelled cells* | *No. of cells in proliferating zone* | *Labelling index (%)* |
|---|---|---|---|---|---|---|---|---|
| 30 | 1 | Olive oil | 19.6 ± 2.4 | 185.4 ± 24.4 | 10.8 ± 1.1 | 2.6 ± 0.3 | 13.6 ± 0.6 | 20.2 ± 1.0 |
| | 2 | $T_4$ | 80.6 ± 4.0[d] | 364.0 ± 35.6[c] | 22.8 ± 2.0[d] | 5.0 ± 0.4[c] | 19.0 ± 1.0[b] | 28.0 ± 1.6[c] |
| 52 | 1 | Olive oil | 17.8 ± 2.3 | 180.4 ± 30.0 | 10.2 ± 0.9 | 2.9 ± 0.2 | 16.1 ± 1.7 | 18.2 ± 1.1 |
| | 2 | $T_4$ | 84.6 ± 6.9[d] | 356.4 ± 35.9[c] | 24.4 ± 2.7[c] | 6.6 ± 0.5[d] | 23.3 ± 0.9[c] | 28.2 ± 1.6[d] |

[a]For explanation of treatments, see Table I. [b–d]Significance of difference from the value in Group 1: [b]$P < 0.05$. [c]$P < 0.01$, [d]$P < 0.001$.

**Figure 1** Histological classification of gastric adenocarcinomas in MNNG-treated rats. **a**, very well-differentiated adenocarcinoma: **b**, common type of well-differentiated adenocarcinoma: **c**, mucinous well-differentiated adenocarcinoma: **d**, signet-ring cell carcinoma. H & E, × 200. *Bars*, 100 μm.

**Table III** Serum $T_4$ and $T_3$ levels in MNNG-treated rats

| *Experimental week* | *Group no.* | *Treatment*[a] | *Thyroid hormone* $T_4$ ($\mu g\ dl^{-1}$) | $T_3$ ($ng\ ml^{-1}$) |
|---|---|---|---|---|
| 30 | 1 | Olive oil | 1.9 ± 0.1 | 0.4 ± 0.0 |
| | 2 | $T_4$ | 16.0 ± 1.1[b] | 2.0 ± 0.0[b] |
| 52 | 1 | Olive oil | 1.8 ± 0.1 | 0.4 ± 0.0 |
| | 2 | $T_4$ | 17.0 ± 1.3[b] | 2.2 ± 0.2[b] |

[a]For explanation of treatments, see Table I. [b]Significance of difference from the value of Group 1 at $P < 0.001$.

The mechanism of this effect is not known, but three possible explanations may be considered. One is an immunomodulatory role of $T_4$. Thyroid hormones have immunostimulatory (Pierpaoli *et al.*, 1969; Bachman & Marshaly, 1986) and immunosuppressive (Gupta *et al.*, 1983) effects on the lymphocyte population. As observed after other endocrine treatments, e.g., with estrogen or cortisone in mice (Milisauskas *et al.*, 1983; Hochman & Cudkowicz, 1979), the inhibition of natural killer cell activity caused by long-term $T_4$ administration might be ascribed to the induction of suppressor cells.

A second possibility is an influence of $T_4$ on the secretion and/or synthesis of regulatory peptides such as epidermal growth factor (EGF) and somatostatin. Walker *et al.* (1981) found that 5 and 10 days treatments with $T_4$ significantly increased the EGF concentration in the submaxillary gland of mice. EGF is a well characterised polypeptide that exhibits mitogenic effects on a wide range of cell types after binding to specific transmembrane receptors (Cohen, 1983). As EGF can stimulate mucosal growth throughout the gastrointestinal tract, it has been suggested as playing a role in gastrointestinal carcinogenesis. Yasui *et al.* (1990) found that prolonged s.c. administration of EGF significantly increased the incidence of gastric cancers induced by MNNG. A high level of $T_4$ was shown to increase the secretion and hypothalamic content of somatostatin *in vivo* and *in vitro* (Berelowitz *et al.*, 1980). We found previously that prolonged s.c. administration of somatostatin significantly increased the incidence and number of gastric cancers (Tatsuta *et al.*, 1989).

A third possibility is an acceleration of cell proliferation by $T_4$. de Launoit and Kiss (1989) found that $T_4$ dramatically stimulated the cell division by MXT (mouse) and MCF-7 (human) mammary cancer cell lines. In mammary tumorigenesis, $T_4$ may exert its stimulatory effect at the level of the nuclear chromatin receptors (Oppenheimer, 1979). In the present work, we found that administration of $T_4$ significantly increased the number of cells in the zone of proliferating cells and the labelling index of the antral and fundic epithelial cells.

Adeniyi and Olowookorun (1989) found that chronic $T_4$ administration significantly increased the mucosal thickness and volume, the parietal cell count per unit mass of the glandular stomach. However, they did not examine the effects of $T_4$ on the antral mucosa. Recently, we examined the effects of long-term administration of $T_4$ for 20 weeks on the fundic and antral mucosa in rats without MNNG pretreatment and found that $T_4$ significantly increased the labelling indices in both the antral (25.0 ± 1.1 *vs* 16.6 ± 1.4%, $P < 0.01$) and the fundic (17.8 ± 1.1 *vs* 10.4 ± 0.5%, $P < 0.001$) mucosa, compared to those in control group. However, we observed neither significant differences between the average heights of the antral and fundic mucosa in the two groups nor mucosal dysplasia associated with $T_4$ treatment in non-carcinogen treated rats.

The present study showed that $T_4$ administration increased the yield of gastric cancers after MNNG treatment, presumably by acting as a non-genotoxic growth stimulator for the mucosal cells. Watanabe *et al.* (1992) found that oral administration of NaCl after MNNG pretreatment significantly increased the incidence of gastric cancers and that it also significantly increased the height of the pyloric mucosa. Many investigators (Takahashi *et al.*, 1986; Tatsuta *et al.*, 1988*a*; Kobori *et al.*, 1984) have indicated that enhancing effects on gastric carcinogenesis may be related to stimulation of cell proliferation and elongation of the mucosa. These findings suggest that a non-genotoxic component increases tumour yield when administered subsequent to a genotoxic agent.

**Abbreviations:**
MNNG, N-methyl-N′-nitro-N-nitrosoguanidine; BrdU, bromodeoxyuridine; $T_4$, L-thyroxine; $T_3$, triiodothyronine; EGF, epidermal growth factor.

### References

ADENIYI, K.O. & OLOWOOKORUN, M.O. (1989). Gastric acid secretion and parietal cell mass: effects of thyroidectomy and thyroxine. *Am. J. Physiol.*, **256**, G975–G978.

BACHMAN, S.E. & MASHALY, M.M. (1986). Relationship between circulating thyroid hormones and humoral immunity in immature male chickens. *Dev. Comp. Immunol.*, **10**, 395–403.

BERELOWITZ, M., MAEDA, K., HARRIS, S. & FROHMAN, L.A. (1980). The effects of alterations in the pituitary-thryoid axis on hypothalamic content in *in vitro* release of somatostatin-like immunoreactivity. *Endocrinology*, **107**, 24–29.

COHEN, S. (1983). The epidermal growth factor (EGF). *Cancer*, **51**, 1787–1791.

DE LAUNOIT, Y. & KISS, R. (1989). Influence of L-thyroxine, L-triiodothyronine, thyroid stimulating hormone, or estradiol on the cell kinetics of cultured mammary cancer cells. *In Vitro Cell Dev. Biol.*, **25**, 585–591.

EASTWOOD, G.L. & QUIMBY, G.F. (1983). Effect of chronic cimetidine ingestion on fundic and antral epithelial proliferation in the rat. *Dig. Dis. Sci.*, **28**, 61–64.

GRATZNER, H.G. (1982). Monoclonal antibody to 5-bromo- and 5-iododeoxyuridine: a new reagent for detection of DNA replication. *Science*, **218**, 474–475.

GUPTA, M.K., CHIANG, T. & DEODHAR, S.D. (1983). Effect of thyroxine on immune response in C57Bl/6J mice. *Acta Endocrinol.*, **103**, 76–80.

HERNANDEZ, D.E., WALKER, C.H. & MASON, G.A. (1988). Influence of thyroid states on stress gastric ulcer formation. *Life Sci.*, **42**, 1757–1764.

HOCHMAN, P.S. & CUDKOWICZ, G. (1979). Suppression of natural cytotoxicity by spleen cells of hydrocortisone-treated mice. *J. Immunol.*, **123**, 968–976.

IISHI, H., TATSUTA, M., BABA, M., OKUDA, S. & TANIGUCHI, H. (1992). Enhancement by thyroxine of experimental carcinogenesis in rat colon induced by azoxymethane. *Int. J. Cancer*, **50**, 974–976.

KOBORI, O., WATANABE, J., SHIMIZU, M. & MORIOKA, Y. (1984). Enhancing effect of sodium taurocholate on N-methyl-N′-nitro-N-nitrosoguanidine-induced stomach tumorigenesis in rats. *Gann*, **75**, 651–654.

MILISAUSKAS, V.K., CUDKOWICZ, G. & NAKAMURA, I. (1983). Role of suppressor cells in the decline of natural killer cell activity in estrogen-treated mice. *Cancer*, **43**, 5240–5243.

MILLER, G.R. Jr (1966). *Simultaneous Statistical Inference*. McGraw-Hill: New York.

MORSTYN, G., HSU, S.M., KINSELLA, T., GRATZNER, H.G., RUSSO, A. & MITCHELL, J.B. (1983). Bromodeoxyuridine in tumors and chromosomes detected with a monoclonal antibody. *J. Clin. Invest.*, **72**, 1844–1850.

OPPENHEIMER, J.H. (1979). Thyroid hormone action at the cellular level. *Science*, **203**, 971–979.

PIERPAOLI, W., BARONI, C., FABRIS, N. & SORKIN, E. (1969). Hormones and immunological capacity. II. Reconstitution of antibody production in hormonally deficient mice by somatotropic hormone, thyrotropic hormone and thyroxin. *Immunology*, **16**, 217–230.

SIEGEL, S. (1956). *Non-Parametric Statistics for the Behavioral Sciences*. McGraw-Hill: New York.

SNEDECOR, G.W. & COCHRAN, W.G. (1967). *Statistical Methods*. Iowa State University Press: Ames, IA.

STEFANINI, M., DE MARTINO, C. & ZAMBONI, L. (1967). Fixation of ejaculated spermatozoa for electron microscopy. *Nature*, **216**, 173–174.

TAKAHASHI, M., HASEGAWA, R., FURUKAWA, F., TOYODA, K., SATO, H. & HAYASHI, Y. (1986). Effects of ethanol, potassium metabisulfite, formaldehyde and hydrogen peroxide on gastric carcinogenesis in rats after initiation with N-methyl-N′-nitro-N-nitrosoguanidine. *Jpn. J. Cancer Res.*, **77**, 118–124.

TATSUTA, M., IISHI, H., BABA, M. & TANIGUCHI, H. (1989). Enhancement by somatostatin of experimental gastric carcinogenesis induced by N-methyl-N′-nitro-N-nitrosoguanidine in Wistar rats. *Cancer Res.*, **49**, 5534–5536.

TATSUTA, M., IISHI, H., BABA, M., YAMAMURA, H. & TANIGUCHI, H. (1988*a*). Enhancement by prolonged administration of caerulein of experimental carcinogenesis induced by N-methyl-N′-nitro-N-nitrosoguanidine in rat stomach. *Cancer Res.*, **48**, 6332–6335.

TATSUTA, M., IISHI, H., YAMAMURA, H., BABA, M., YAMAMOTO, R. & TANIGUCHI, H. (1988*b*). Effects of cimetidine on inhibition by tetragastrin of gastric carcinogenesis induced by N-methyl-N′-nitro-N-nitrosoguanidine in Wistar rats. *Cancer Res.*, **48**, 1591–1595.

WALKER, P., WEICHSEL, M.E. Jr, HOATH, S.R., POLAND, R.E. & FISHER, D.A. (1981). Effect of thyroxine, testosterone, and corticosterone on nerve growth factor (NGF) and epidermal growth factor (EGF) concentrations in adult female mouse submaxillary gland: dissociation of NGF and EGF response. *Endocrinology*, **109**, 582–587.

WATANABE, H., TAKAHASHI, T., OKAMOT, T., OGUNDIGIE, P.O. & ITO, A. (1992). Effects of sodium chloride and ethanol on stomach tumorigenesis in ACI rats treated with N-methyl-N′-nitro-N-nitrosoguanidine: a quantitative morphmetric approach. *Jpn. J. Cancer*, **83**, 588–593.

YASUI, W., TAKEKURA, N., KAMADA, T., ODA, N., ITO, M., ITO, H. & TAHARA, E. (1990). Effect of epidermal growth factor on rat stomach carcinogenesis induced by N-methyl-N′-nitro-N-nitrosoguanidine. *Acta Pathol. Jpn.*, **40**, 165–171.


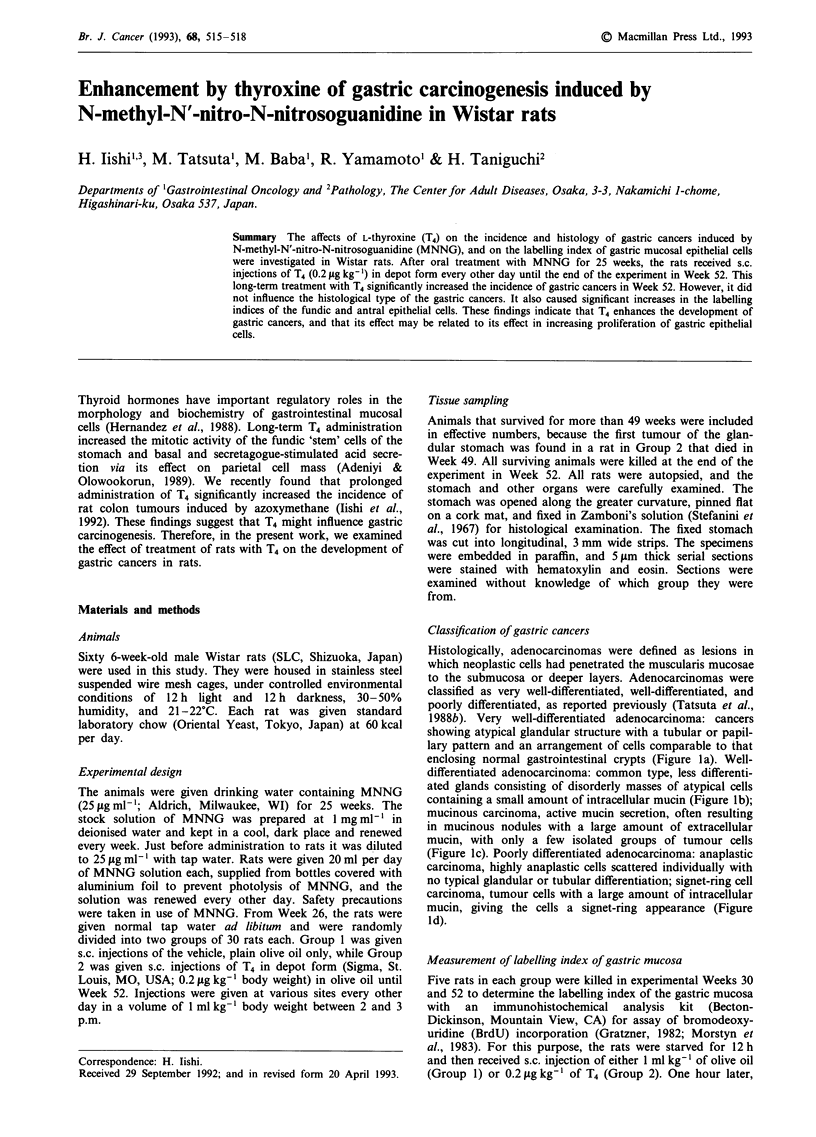

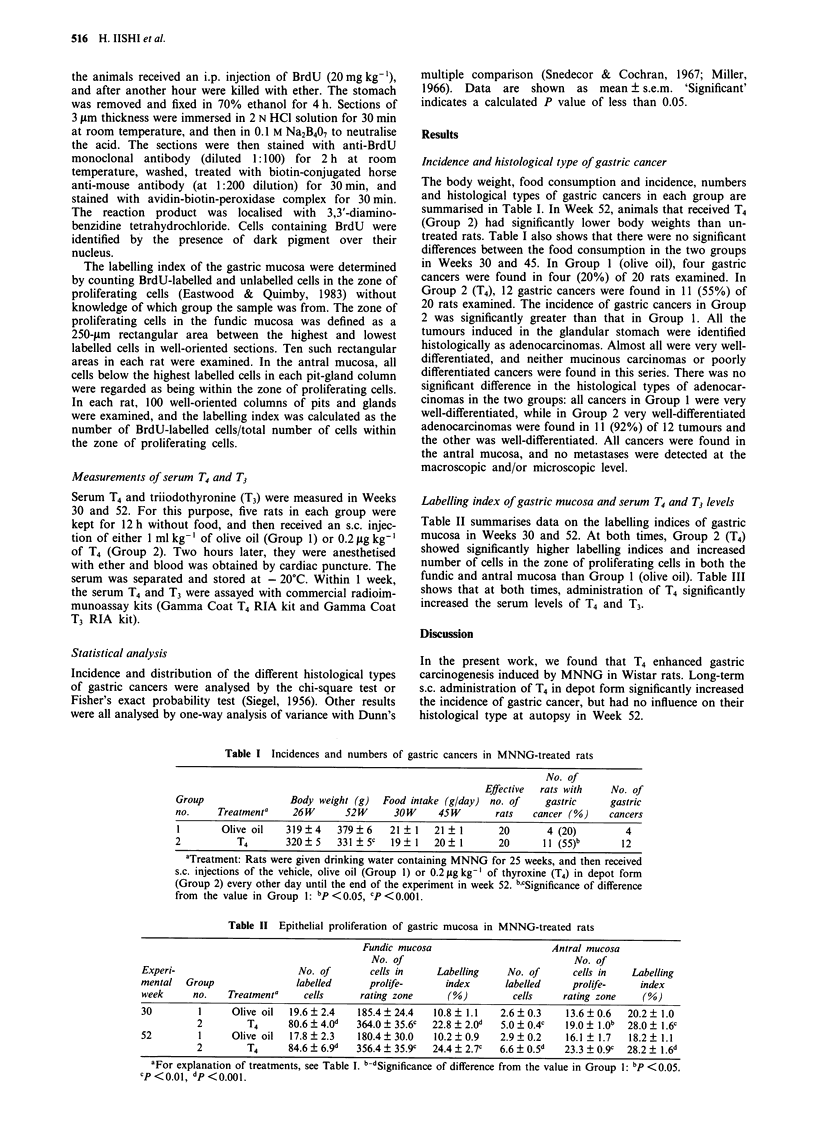

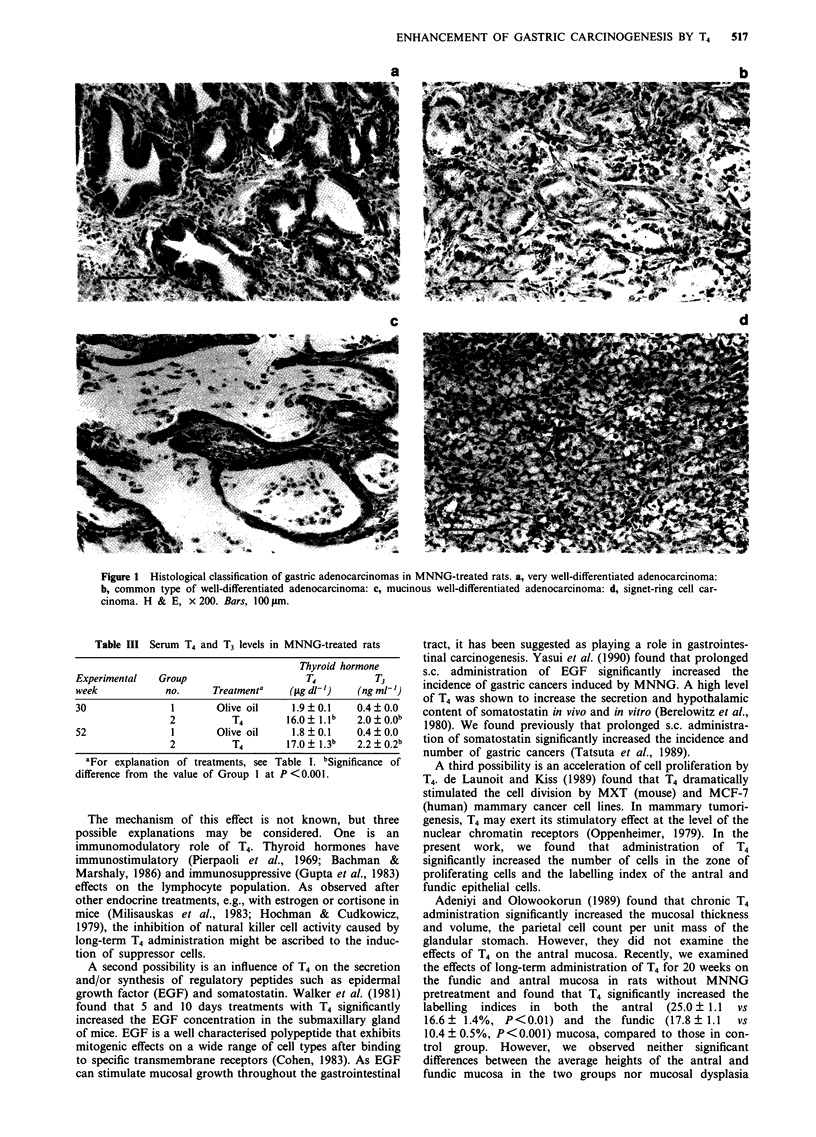

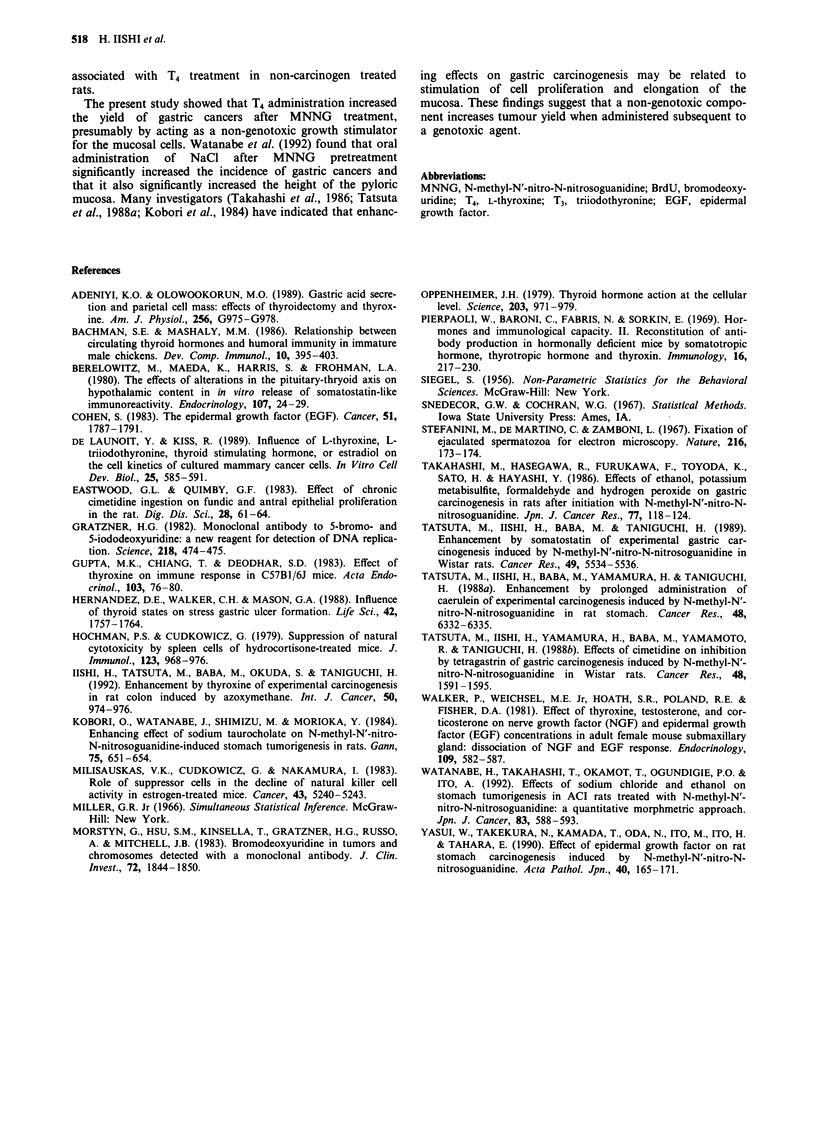


## References

[OCR_00407] Adeniyi K. O., Olowookorun M. O. (1989). Gastric acid secretion and parietal cell mass: effects of thyroidectomy and thyroxine.. Am J Physiol.

[OCR_00412] Bachman S. E., Mashaly M. M. (1986). Relationship between circulating thyroid hormones and humoral immunity in immature male chickens.. Dev Comp Immunol.

[OCR_00417] Berelowitz M., Maeda K., Harris S., Frohman L. A. (1980). The effect of alterations in the pituitary-thyroid axis on hypothalamic content and in vitro release of somatostatin-like immunoreactivity.. Endocrinology.

[OCR_00423] Cohen S. (1983). The epidermal growth factor (EGF).. Cancer.

[OCR_00433] Eastwood G. L., Quimby G. F. (1983). Effect of chronic cimetidine ingestion on fundic and antral epithelial proliferation in the rat.. Dig Dis Sci.

[OCR_00438] Gratzner H. G. (1982). Monoclonal antibody to 5-bromo- and 5-iododeoxyuridine: A new reagent for detection of DNA replication.. Science.

[OCR_00443] Gupta M. K., Chiang T., Deodhar S. D. (1983). Effect of thyroxine on immune response in C57Bl/6J mice.. Acta Endocrinol (Copenh).

[OCR_00448] Hernandez D. E., Walker C. H., Mason G. A. (1988). Influence of thyroid states on stress gastric ulcer formation.. Life Sci.

[OCR_00453] Hochman P. S., Cudkowicz G. (1979). Suppression of natural cytotoxicity by spleen cells of hydrocortisone-treated mice.. J Immunol.

[OCR_00458] Iishi H., Tatsuta M., Baba M., Okuda S., Taniguchi H. (1992). Enhancement by thyroxine of experimental carcinogenesis induced in rat colon by azoxymethane.. Int J Cancer.

[OCR_00464] Kobori O., Watanabe J., Shimizu T., Shoji M., Morioka Y. (1984). Enhancing effect of sodium taurocholate on N-methyl-N'-nitro-N-nitrosoguanidine-induced stomach tumorigenesis in rats.. Gan.

[OCR_00470] Milisauskas V. K., Cudkowicz G., Nakamura I. (1983). Role of suppressor cells in the decline of natural killer cell activity in estrogen-treated mice.. Cancer Res.

[OCR_00479] Morstyn G., Hsu S. M., Kinsella T., Gratzner H., Russo A., Mitchell J. B. (1983). Bromodeoxyuridine in tumors and chromosomes detected with a monoclonal antibody.. J Clin Invest.

[OCR_00485] Oppenheimer J. H. (1979). Thyroid hormone action at the cellular level.. Science.

[OCR_00489] Pierpaoli W., Baroni C., Fabris N., Sorkin E. (1969). Hormones and immunological capacity. II. Reconstitution of antibody production in hormonally deficient mice by somatotropic hormone, thyrotropic hormone and thyroxin.. Immunology.

[OCR_00504] Stefanini M., De Martino C., Zamboni L. (1967). Fixation of ejaculated spermatozoa for electron microscopy.. Nature.

[OCR_00509] Takahashi M., Hasegawa R., Furukawa F., Toyoda K., Sato H., Hayashi Y. (1986). Effects of ethanol, potassium metabisulfite, formaldehyde and hydrogen peroxide on gastric carcinogenesis in rats after initiation with N-methyl-N'-nitro-N-nitrosoguanidine.. Jpn J Cancer Res.

[OCR_00516] Tatsuta M., Iishi H., Baba M., Taniguchi H. (1989). Enhancement by somatostatin of experimental gastric carcinogenesis induced by N-methyl-N'-nitro-N-nitrosoguanidine in Wistar rats.. Cancer Res.

[OCR_00522] Tatsuta M., Iishi H., Baba M., Yamamura H., Taniguchi H. (1988). Enhancement by prolonged administration of caerulein of experimental carcinogenesis induced by N-methyl-N'-nitro-N-nitrosoguanidine in rat stomach.. Cancer Res.

[OCR_00529] Tatsuta M., Iishi H., Yamamura H., Baba M., Yamamoto R., Taniguchi H. (1988). Effect of cimetidine on inhibition by tetragastrin of carcinogenesis induced by N-methyl-N'-nitro-N-nitrosoguanidine in Wistar rats.. Cancer Res.

[OCR_00536] Walker P., Weichsel M. E., Hoath S. B., Poland R. E., Fisher D. A. (1981). Effect of thyroxine, testosterone, and corticosterone on nerve growth factor (NGF) and epidermal growth factor (EGF) concentrations in adult female mouse submaxillary gland: dissociation of NGF and EGF responses.. Endocrinology.

[OCR_00544] Watanabe H., Takahashi T., Okamoto T., Ogundigie P. O., Ito A. (1992). Effects of sodium chloride and ethanol on stomach tumorigenesis in ACI rats treated with N-methyl-N'-nitro-N-nitrosoguanidine: a quantitative morphometric approach.. Jpn J Cancer Res.

[OCR_00551] Yasui W., Takekura N., Kameda T., Oda N., Ito M., Ito H., Tahara E. (1990). Effect of epidermal growth factor on rat stomach carcinogenesis induced by N-methyl-N'-nitro-N-nitrosoguanidine.. Acta Pathol Jpn.

[OCR_00427] de Launoit Y., Kiss R. (1989). Influence of L-thyroxine, L-triiodothyronine, thyroid stimulating hormone, or estradiol on the cell kinetics of cultured mammary cancer cells.. In Vitro Cell Dev Biol.

